# Improving Voice Outcomes After Thyroid Surgery – Review of Safety Parameters for Using Energy-Based Devices Near the Recurrent Laryngeal Nerve

**DOI:** 10.3389/fendo.2021.793431

**Published:** 2021-11-24

**Authors:** Jia Joanna Wang, Tzu-Yen Huang, Che-Wei Wu, Yi-Chu Lin, Hsin-Yi Tseng, Cheng-Hsin Liu, I-Cheng Lu, Pi-Ying Chang, Hui-Chun Chen, Hsiu-Ya Chen, Gianlorenzo Dionigi, Feng-Yu Chiang, Ling-Feng Wang

**Affiliations:** ^1^ Department of Otorhinolaryngology-Head and Neck Surgery, International Thyroid Surgery Center, Kaohsiung Medical University Hospital, Kaohsiung Medical University, Kaohsiung, Taiwan; ^2^ Department of Otolaryngology-Head and Neck Surgery, Kaohsiung Municipal Siaogang Hospital, Kaohsiung Medical University, Kaohsiung, Taiwan; ^3^ Department of Biological Science and Technology, National Yang Ming Chiao Tung University, Hsinchu, Taiwan; ^4^ Center for Liquid Biopsy and Cohort Research, and Faculty of Medicine, College of Medicine, Kaohsiung Medical University, Kaohsiung, Taiwan; ^5^ Department of Anesthesiology, Kaohsiung Municipal Siaogang Hospital, Kaohsiung Medical University, Kaohsiung, Taiwan; ^6^ Department of Anesthesiology, Kaohsiung Municipal Tatung Hospital, Kaohsiung Medical University, Kaohsiung, Taiwan; ^7^ Department of Nursing, Kaohsiung Medical University Hospital, Kaohsiung, Taiwan; ^8^ Department of Anesthesiology, Kaohsiung Medical University Hospital, Kaohsiung, Taiwan; ^9^ Division of General Surgery, Endocrine Surgery Section, Istituto Auxologico Italiano IRCCS, Milan, Italy; ^10^ Department of Pathophysiology and Transplantation, University of Milan, Milan, Italy; ^11^ Department of Otolaryngology-Head and Neck Surgery, E-Da Hospital, Kaohsiung, Taiwan; ^12^ School of Medicine, College of Medicine, I-Shou University, Kaohsiung, Taiwan

**Keywords:** energy-based devices, safety parameter, recurrent laryngeal nerve, thyroid surgery, voice

## Abstract

Technological advances in thyroid surgery have rapidly increased in recent decades. Specifically, recently developed energy-based devices (EBDs) enable simultaneous dissection and sealing tissue. EBDs have many advantages in thyroid surgery, such as reduced blood loss, lower rate of post-operative hypocalcemia, and shorter operation time. However, the rate of recurrent laryngeal nerve (RLN) injury during EBD use has shown statistically inconsistent. EBDs generate high temperature that can cause iatrogenic thermal injury to the RLN by direct or indirect thermal spread. This article reviews relevant medical literatures of conventional electrocauteries and different mechanisms of current EBDs, and compares two safety parameters: safe distance and cooling time. In general, conventional electrocautery generates higher temperature and wider thermal spread range, but when applying EBDs near the RLN adequate activation distance and cooling time are still required to avoid inadvertent thermal injury. To improve voice outcomes in the quality-of-life era, surgeons should observe safety parameters and follow the standard procedures when using EBDs near the RLN in thyroid surgery

## Introduction

Thyroidectomy is one of the most common head and neck surgeries. In the United States, more than 150,000 thyroidectomies were performed annually, and the case number of thyroid surgery is increasing each year (1). Specific challenges of thyroid surgery include the complex vascularization of anatomy, the proximity of the operating field to the recurrent laryngeal nerve (RLN), and the need to preserve the parathyroid gland. Therefore, thorough hemostasis is essential for avoiding further complications after thyroid surgery (2).

The “clamp-and-tie” technique was first introduced by Theodor Kocher in the 19th century. Since then, until the 1980s, clamp-and-tie, suture-ligation, electrocautery (monopolar or bipolar) and hemostatic clips have been widely adopted for hemostatic use in thyroid surgery (1, 3, 4). However, one of the most important technological advances in thyroid surgery occurred three decades ago: the introduction of energy-based devices (EBDs). Although the specific purpose of using EBDs in thyroid surgery is to achieve hemostasis, the mechanism through which EBDs achieve hemostasis differs from one devices to another (3).

Recent studies indicate that EBDs are used in 65.7% of thyroidectomy patients (1). Reported intraoperative advantages of EBDs include considerably decreased operative time, incision length and blood loss. Other clinically significant advantages of EBDs over conventional techniques include the superior postoperative outcomes of EBDs, e.g., reduced pain, wound drainage, neck hematoma, and hypocalcemia (1, 2, 5, 6).

In terms of RLN safety, however, EBDs have not proven superior to conventional devices used for thyroid surgery. For example, reported rates of RLN injury showed statistically inconsistent between EBDs and conventional devices (7). In fact, one 10-year meta-analysis reported that EBD use was associated with higher RLN paralysis rates (3). In another study, clinical outcomes of thyroidectomy were compared between 11,355 thyroidectomy patients treated with EBDs and a control group treated without EBDs. Compared to the non-EBD group, the EBD group had a significantly higher rate of hoarseness. However, the rate of severe hoarseness was significantly higher in the non-EBD group (1, 8, 9).

In another study, Liu et al. reported that the RLN palsy rate did not significantly differ between an EBD group and a clamp-and-tie group. However, the nerve injury mechanisms are significantly differed between the two groups. No patients in the clamp-and-tie group had thermal injury, whereas one-third of patients in the EBD group suffered thermal injury caused by lateral thermal spread in palsied RLNs (6).

## Intraoperative Neuromonitoring and RLN Thermal Injury Caused by EBDs

Intraoperative injury to the RLN can cause vocal cord paralysis. Symptoms may include hoarseness, choking, dysphagia and dysphonia in unilateral vocal cord paralysis, in addition, difficulty breathing may occur in cases of bilateral vocal cord paralysis (10–13). To reduce this major cause of morbidity after thyroid surgery, visual identification of RLN is the standard practice, and IONM facilitates this procedure (10, 14–16). Use of IONM not only enables the surgeons to evaluate RLN function in real time, but also to assess the mechanism of an impending or actual RLN injury and the appropriate surgical procedure for preventing or treating the injury (17–21). By elucidating the mechanism of iatrogenic RLN injury with IONM, many studies noted that traction and thermal injury are the first and second most common causes of RLN injury during thyroidectomy (5, 17, 22). Most intraoperative thermal injuries to the RLN result from thermal spread during use of high-temperature electrocautery devices and EBDs. Tissue contraction during EBD activation increases the risk of thermal injury because it reduces the distance from the RLN at which an EBD can be safely used (6). Thermal injury to the RLN often occurs unexpectedly when an EBD is activated or used for dissection. This type of injury usually is not only severe but irreversible (17).

**Figure 1:**
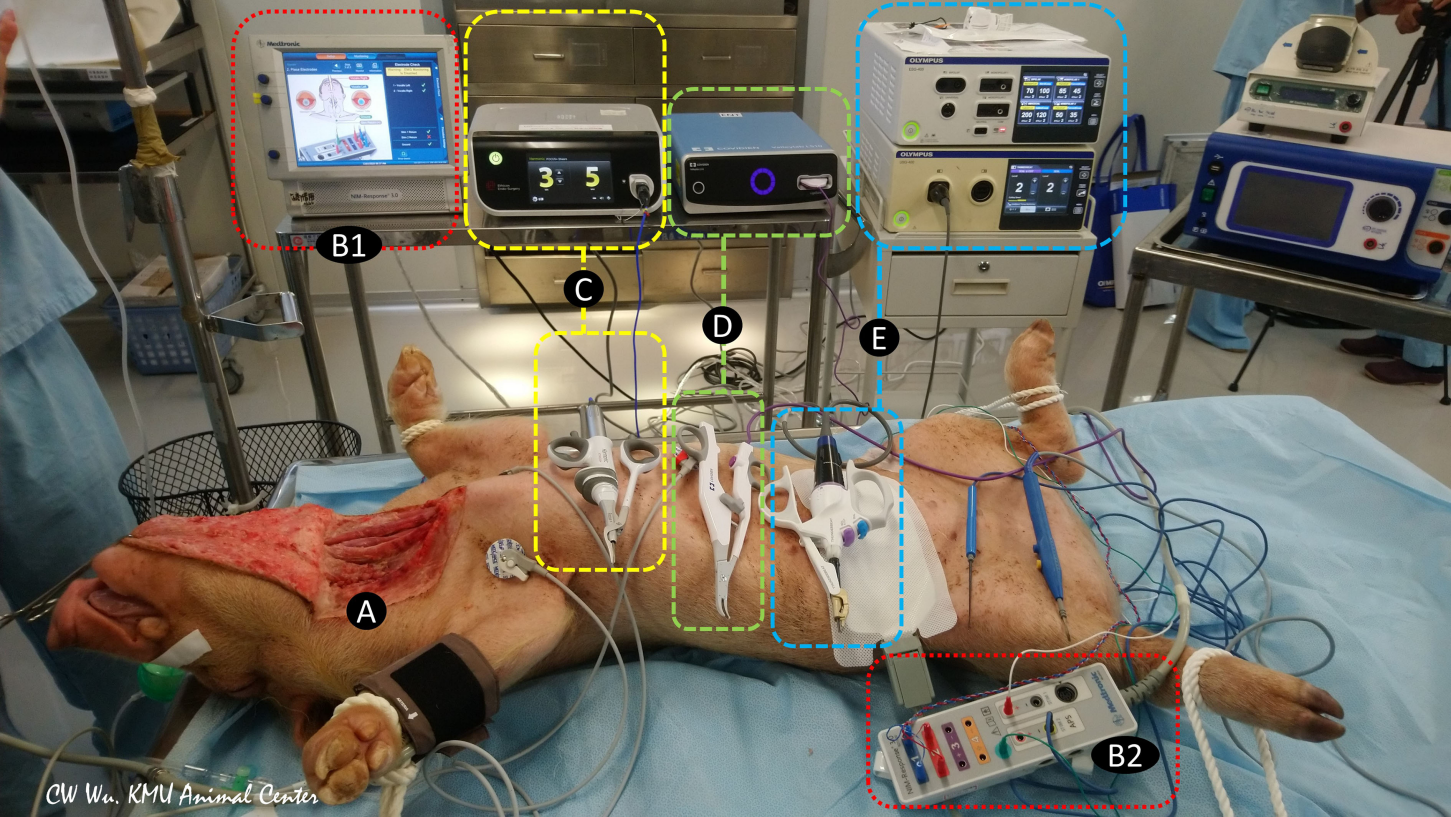
Animal models of continuous intraoperative neuromonitoring (cIONM) provide an objective platform for optimizing the safety parameters for various energy-based devices used for hemostasis and dissection near the recurrent laryngeal nerve (RLN) during thyroid surgery. **(A)** Preparation of porcine animal model by transverse incision and flap formation in neck skin. **(B1)** IONM monitoring system used for recording, monitoring, and analyzing real-time changes in laryngeal EMG. **(B2)** IONM interface box for connecting recording, stimulation, and ground electrodes. **(C)** Ultrasonic energy based device and energy generator. **(D)** Advanced biopolar energy based device and energy platform. **(E)** Hybrid energy based device (ultrasonic and bipolar energy) and multifunctional platform.

In addition to the conventional uses of EBDs for tissue sealing, cutting, and hemostasis, surgeons have recently begun using EBDs for grasping and dividing tissue when dissection is performed near the RLN or even in direct contact with the RLN, especially in endoscopic procedures. As a results of technological advances in EBDs and the growing acceptance of surgical applications of EBDs, the increased incidence of thermal injury has emerged one of the main adverse events in thyroid surgery (23). Direct thermal burn injury can occur during or after activation of an EBD when the temperature of the blade is still high. Such injuries result from inadvertent contact between the blade and the skin or surrounding soft tissue, especially the RLN (24). Unlike direct thermal injury to the RLN, an indirect thermal injury to the RLN, which results from lateral thermal spread, is rarely visible to the naked eye. Therefore, animal experiments by using continuous IONM are needed for objective evaluation of the electrophysiology of RLN thermal injury and for establishing guidelines for safe use of EBDs, including minimum cooling time and safe distance between the EBD tip and the skin or surrounding soft tissue (**Figure 1**) (25, 26).

## Animal Studies in EBD Safety

**Figure 2:**
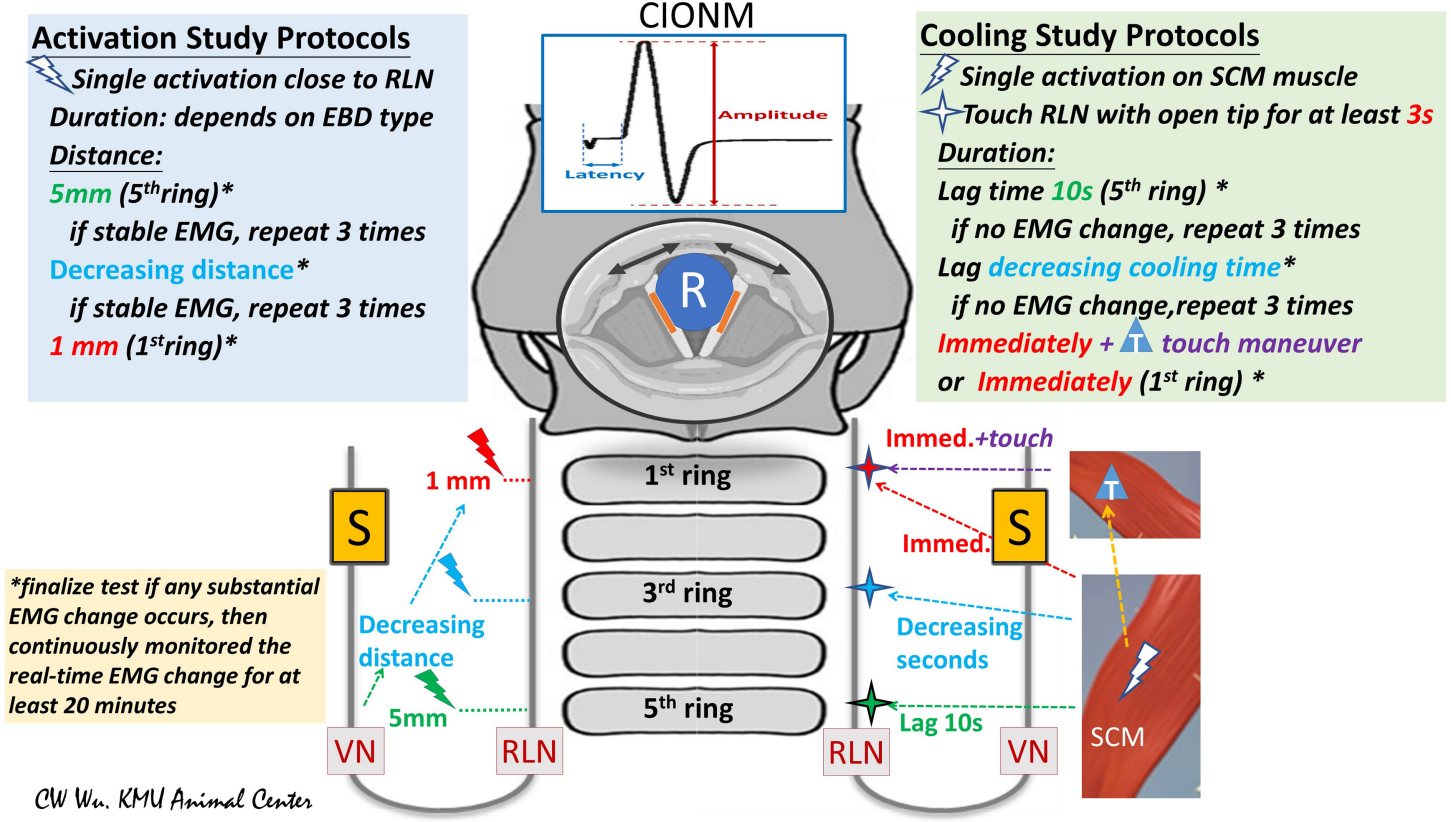
Flowchart of study protocols for using energy-based devices (EBDs). **(Left)** Activation Study Protocols: Tests were performed from the proximal to distal segments of the RLN. The distance from the tip of the EBD to the RLN was measured. In this study, the first test was performed at a distance of 5 mm from the fifth tracheal ring. If the EMG remained stable after three tests, further tests were performed at a shorter distance. If the EMG remained stable after repeated tests, further tests were performed at a distance of 1 mm or with the EBD tip in direct contact with the RLN. **(Right)** Cooling Study Protocol: Tests were performed on the RLN from the proximal to distal segment. After a single EBD activation on the SCM muscle, the operator touched the RLN with the tip of the open blade after varying cooling times. The fifth tracheal ring was touched after a cooling time of 10 seconds. If EMG remained stable in three tests, tests proceeded from the proximal RLN (fifth tracheal ring, green cross) to the distal RLN (first tracheal ring, red cross) after progressively decreasing cooling times. *asterisk: If a substantial EMG change was noted, the RLN experiment was considered complete, and EMG was continuously monitored for at least 20 minutes. T, touch maneuver: cooling by quickly touching surrounding tissue. S, Stimulation electrode for automatic periodic stimulation (APS) of vagus nerve (VN); R, Recording electrode on endotracheal tube for recording electromyography (EMG) signals evoked by vocal cord movement (black arrow); cIONM, continuous intraoperative neuromonitoring; RLN, recurrent laryngeal nerve; VN, vagus nerve; EBD, energy-based device; SCM, sternocleidomastoid; EMG, electromyography.

Studies of EBD safety in the literature are typically performed in two stages: activation studies and cooling studies. Activation studies assess the distance from the RLN at which an EBD can be safely used without causing thermal injury; cooling studies assess the time (after activation) needed for the EBD tip or blade to cool sufficiently for use of the EBD in performing a dissection close to or in contact with the RLN (**Figure 2**).

### Activation Studies

The EBD is typically applied to RLN soft tissue at a distance starting from 5 mm and gradually decreased to 0 mm. Real-time electrophysiologic electromyography (EMG) information is continuously recorded, compared and analyzed during varying durations of activation and under varying power settings. If a substantial EMG change occurs after any test, the RLN experiment is stopped, and real-time EMG is continuously recorded for 20 to 60 minutes to determine whether the injury is reversible (**Figure 2**, left) (5, 25, 27).

### Cooling Studies

After activation of the EBD on the sternocleidomastoid (SCM) muscle, surgeons touches the tip on the RLN after 10 seconds of waiting and cooling, then the cooling time gradually decreases to 0 seconds and observes the EMG for adverse changes. The “muscle touch maneuver” can be performed by touching the EBD tip/blade to the surrounding tissue right after activation. This maneuver offers ideal cooling effect by reducing the temperature immediately (**Figure 2**, right) (25).

## Electrocauteries and Energy-Based Devices

### Monopolar Electrocautery

Electrocautery equipment is most commonly used in conventional thyroidectomy. In a monopolar electrocautery, current from the probe electrode passes through the patient to a return pad. This equipment is known for its lateral and vertical transmission and diffusion of electrical power into surrounding tissue. Since monopolar electrocautery rapidly generates temperatures exceeding 350°C, thermal spread in tissues is problematic (27–29). Wu et al. reported that safe use of monopolar electrocautery at 15 watts requires an activation distance of 5 mm and a cooling time of 1 second (25). However, the optimal safety parameters (i.e., activation distance and time) depend on the activation power use to on the tissue.

### Bipolar Electrocautery

In bipolar electrocautery, current only passes through tissue between two arms of a forceps-shaped electrode. Bipolar electrocautery is performed at a temperature significantly lower than that of monopolar electrocautery, and showed little difference of Celsius degree when compare to advanced bipolar EBDs (e.g., Ligasure) (28). An animal study indicated that, the safety parameters for bipolar electrocautery performed at 30 watts are an activation distance of 3 mm and a cooling time of 1 second (25).

### Advanced Bipolar EBDs

**Figure 3:**
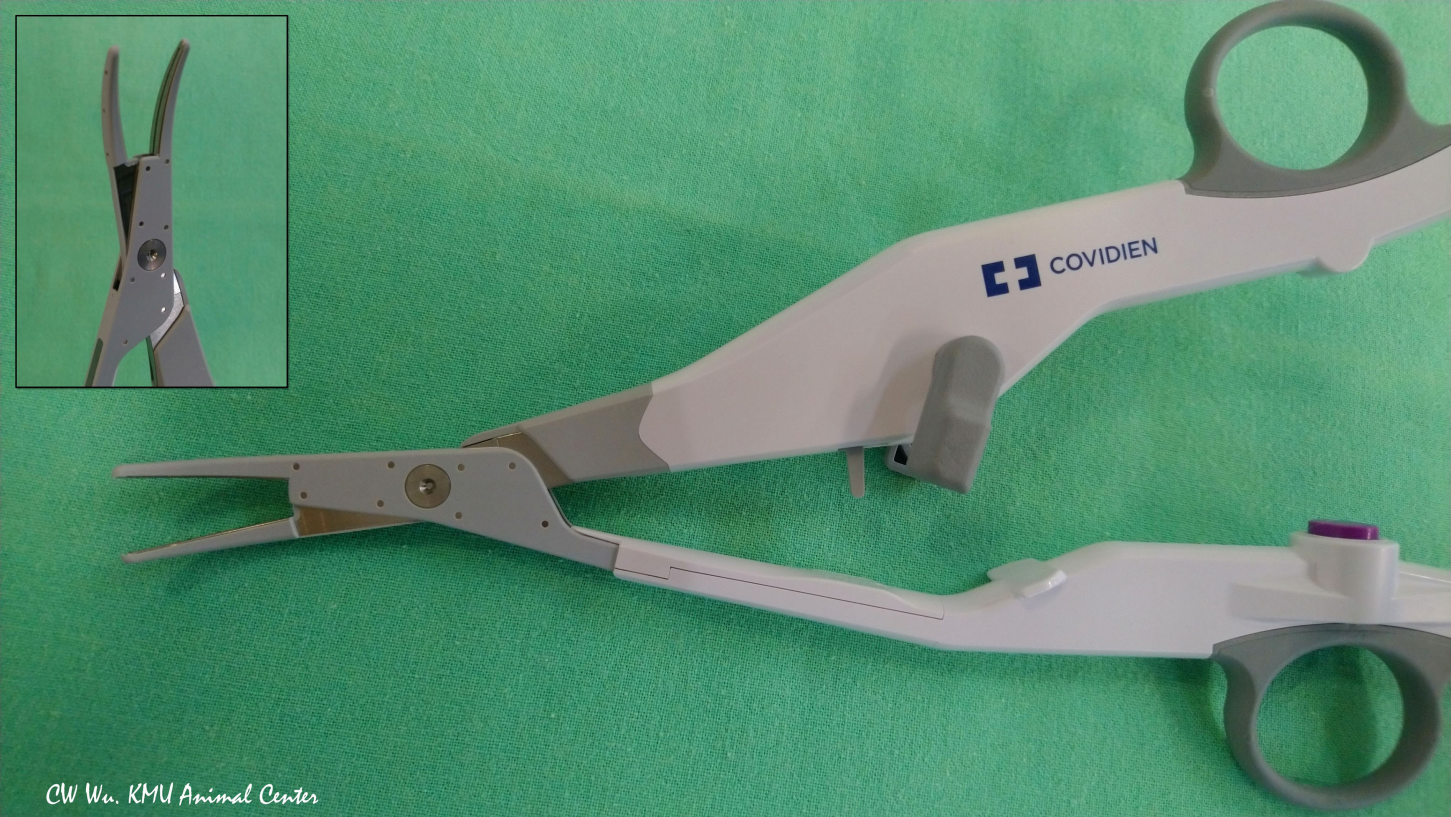
The LigaSure Exact Dissector (LED) (Medtronic, Mineapolis, MN, USA) is an advanced bipolar energy based device commonly used for open thyroid surgery. The finely curved symmetrical jaws have an anti-adherent nano-coating.

LigaSure, an advanced bipolar EBD, can seal vessels up to 7 mm in diameter (30, 31). The Ligasure Small Jaw (LSJ) and Ligasure Exact Dissector (LED) have curved jaws with bilateral symmetric blades coated with anti-adherent material to enable their use in performing fine-manipulation tasks (**Figure 3**). Reports of experiments performed using infrared cameras or thermosensors indicate that both devices operate at a temperature below 100°C (32–34).

**LigaSure Small Jaw (LSJ)** (Medtronic, Minneapolis, MN, USA)

The LSJ has an activation button with tactile feedback and a 16.5 mm long curved tip. According to Dionigi et al, the safe activation distance is 2 mm, and a 2-second interval or muscle touch maneuver is required for cooling (31).

**LigaSure Exact Dissector (LED)** (Medtronic, Minneapolis, MN, USA)

Compared to LSJ, the LED has a narrower jaw (2 mm) and a longer seal (20.6 mm). For sealing, the required activation time is 2 to 4 seconds, which is shorter than that of LSJ. In Huang et al. study, the safe activation distance was 1 mm, and the cooling study revealed no adverse EMG events after a 2-second cooling interval or after muscle touch maneuver (35).

### Ultrasonic EBDs

**Figure 4:**
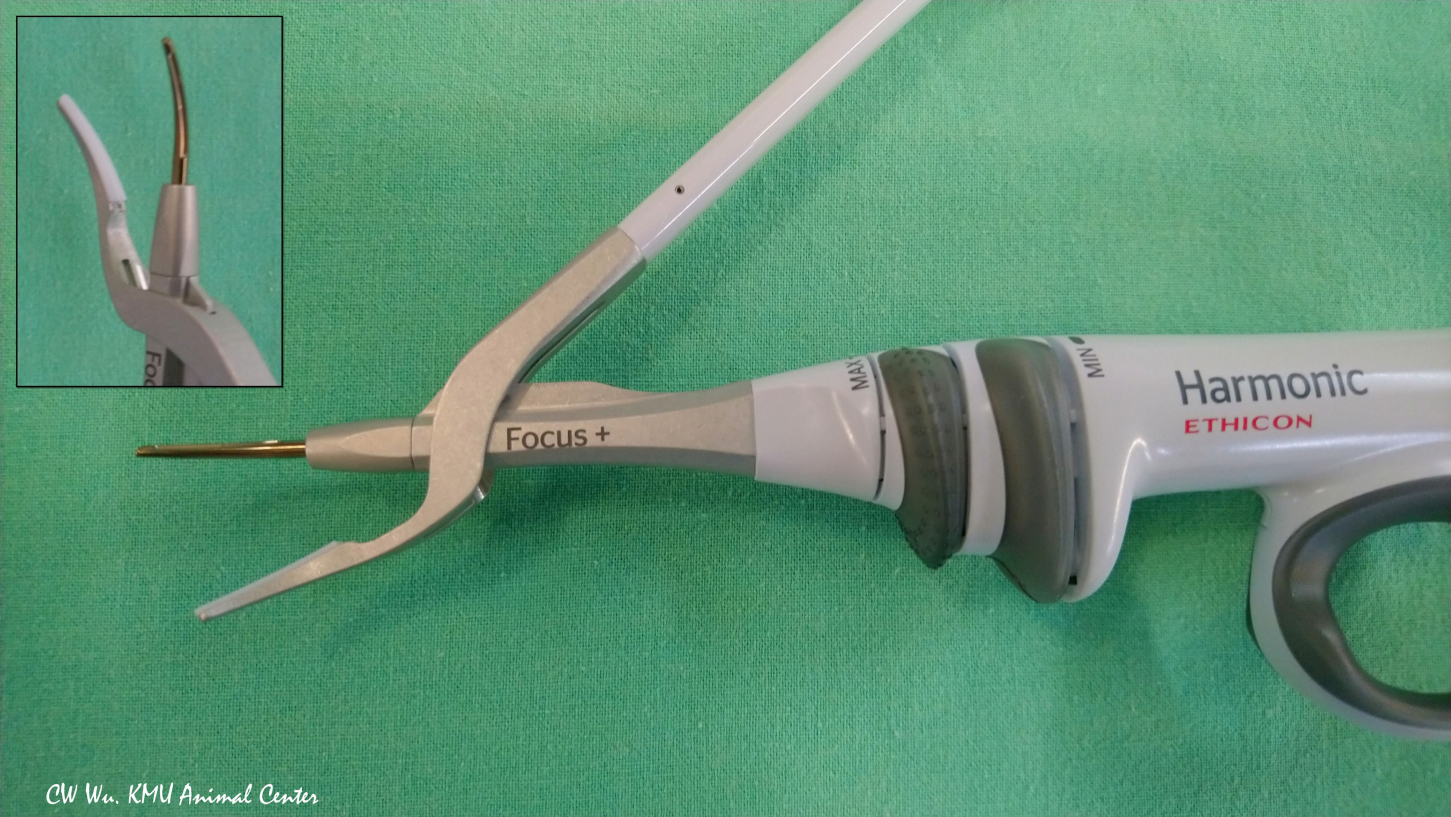
Ultrasonic energy based device. Harmonic Focus+ (HF+) (Ethicon, Johnson and Johnson, Cincinnati, OH, USA). The HF+ is widely used in open thyroid surgery. Its curved and tapered tip has bare blades on one side and a non-active tissue pad on the opposite side.

Ultrasonic EBDs deliver energy in the form of ultrasonic vibrations, which enable simultaneous cutting and coagulation. When a Harmonic device is used for transecting and sealing tissue, contact between the blade and the tissue pad of the device causes a rapid temperature increase.(**Figure 4**) A Harmonic device can seal vessels up to 7 mm in diameter. Notably, ultrasonic EBDs such as the Harmonic enable sealing at lower temperatures compared to monopolar electrocautery equipment (30, 36).

**Harmonic Focus (HF)** (Ethicon, Johnson and Johnson, Cincinnati, OH, USA)

The activation study in Wu et al. revealed no adverse EMG event at an activation distance of 1 mm and an activation time between 3 and 10 seconds. At a distance of 0 mm, EMG was stable in the first 3 seconds, but adverse EMG events occurred when activation time reached 4 seconds or longer. The cooling study revealed no adverse EMG event after a 10-second cooling period. When muscle touch maneuver was used for cooling, no adverse EMG events occurred after a 2-second cooling time (37).

**Harmonic Focus+ (HF+)** (Ethicon, Johnson and Johnson, Cincinnati, OH, USA)

Unlike the HF, the HF+ has Adaptive Tissue Technology, which increases precision in delivery of energy and reduces the time required for the device to reach its operating temperature (36). In the study, the HF+ did not cause significant thermal damage to the RLN at an activation distance of 1mm or even when the non-acting blade was activated while in direct contact with the nerve in the dry field. However, activation at a distance of 1 mm increased latency in some nerves whereas activation in direct contact revealed decreased amplitude in some nerves. In both cases, EMG changes recovered to baseline within 5-6 minutes (27).

**Harmonic ACE** (Ethicon, Johnson and Johnson, Cincinnati, OH, USA)

The Harmonic ACE is applicable in both endoscopic and open surgery. The device, 23cm length, 5.5mm diameter, can rotate 360 degrees. Kim et al. reported no adverse EMG events within 25 seconds after activation at a distance of 4 mm. At activation distances of 1-3 mm, however, shrinkage occurred in adjacent tissue within 6 to 25 seconds after activation. In some patients, activation resulted in adverse EMG events (38).

**Harmonic ACE+** (Ethicon, Johnson and Johnson, Cincinnati, OH, USA)

The Adaptive Tissue Technology used in the HF+ is also used in the Harmonic ACE+. This technology theoretically reduces thermal spread and transection time. Experiments showed that Harmonic ACE+ did not cause adverse EMG events within 20 seconds after activation at a distance of 1 mm from the RLN. When the HA+ was activated in direct contact with the RLN, however, adverse EMG events occurred after 6 seconds of activation (38).

**Sonicision** (Medtronic, Minneapolis, MN, USA)

Sonicision is an ultrasonic EBD that can perform vessel sealing and dissection in surgery. The device is cordless, which increases mobility and convenience. Since the device is available in lengths ranging from 13 to 48 cm, it can be used in either endoscopic or open surgery. According to Hayami et al., Sonicision can be safely used at a distance of 1 mm from the RLN (39).

### Hybrid EBDs (Ultrasonic and Bipolar EBDs)

**Thunderbeat (TB)** (Olympus Co Inc, Tokyo, Japan)

**Figure 5:**
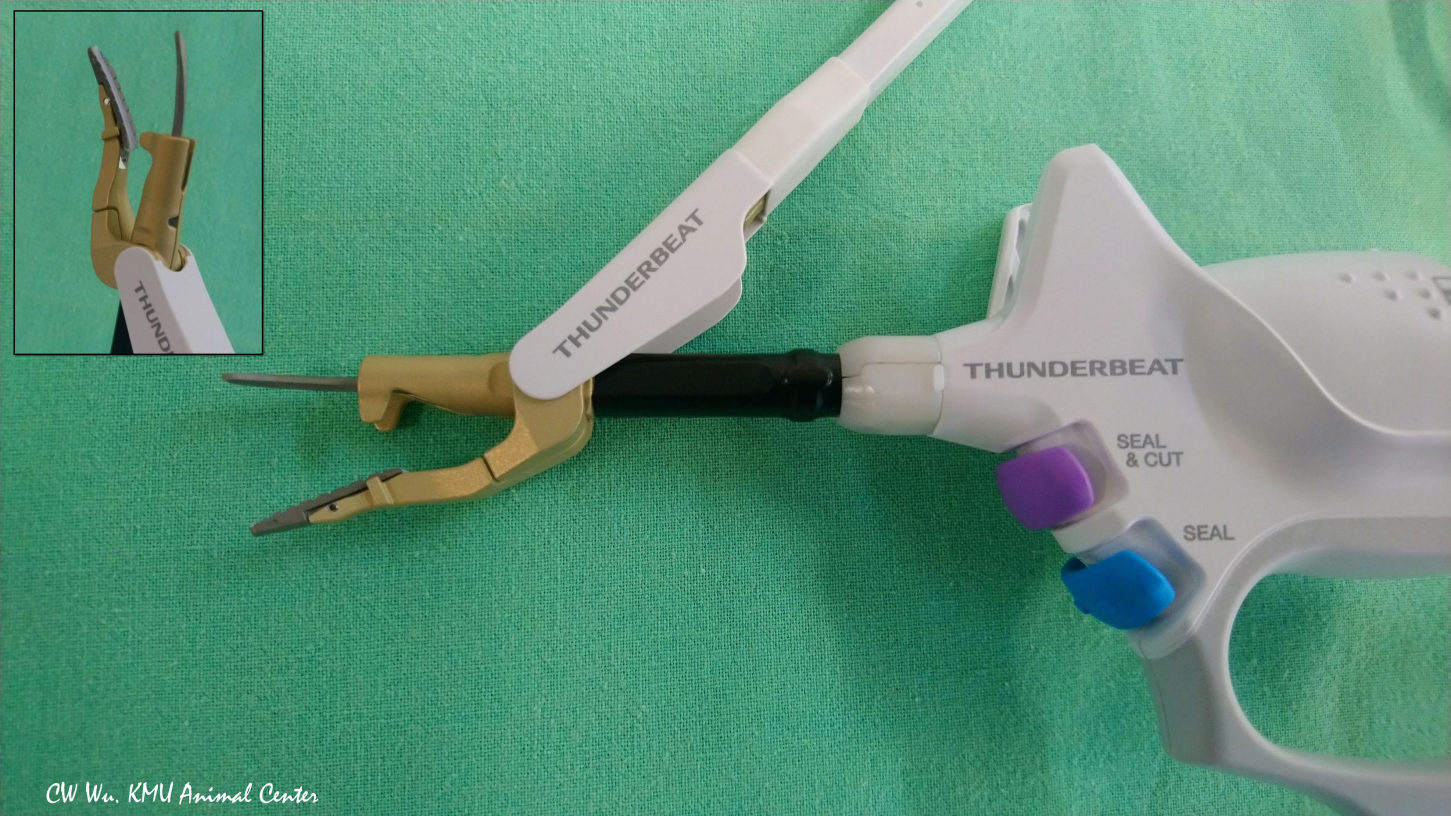
Hybrid energy based device (ultrasonic and bipolar energy). The Thunderbeat Open Fine Jaw (TB; Olympus Co Inc, Tokyo, Japan) has a thin, curved probe for fine dissection. The counter jaw enables its use for grasping tissue. The aluminum coating dissipates residual heat.

The TB integrates both ultrasonic and advanced bipolar energy. The curved probe is composed of aluminum and has a thin profile to aid release of residual heat after activation (**Figure 5**). Surgeons can use TB to coagulate, dissect and cut blood vessels 5-7 mm in diameter (4, 33). In Kwak et al. study, no adverse EMG events occurred when the TB was activated for less than 10 seconds at distances of 3 mm from the RLN. However, when the TB was activated for 8 seconds at a distance of 2 mm from the RLN, amplitude decreased (4).

### Ferromagnetic EBDs

Ferromagnetic EBDs are recently developed devices that enable hemostasis and dissection during thyroid surgery. Ferromagnetic EBDs generate pure thermal energy in response to a rapidly alternating magnetic field (40, 41).

**FMwand** (Domain Surgical, Salt Lake City, Utah)

The FMwand is a hemostatic dissecting scalpel with a dissecting loop. In Huang et al. study, no adverse EMG events occurred at distances of 2 mm or longer. Additionally, the cooling study revealed no adverse EMG events after a 1-second interval or after muscle touch maneuver (40).

**FMsealer** (Domain Surgical, Salt Lake City, Utah)

The FMsealer is a vessel sealing instrument that can seal vessels up to 7 mm in diameter. Compared to ultrasonic devices, FMsealer has a significantly lower peak temperature (92.1°C) and enables faster transection of tissue bundles (41). In their activation study, Huang et al. reported that the safe distance is 2 mm for a single 3-second activation. No adverse EMG events occurred after a 3-second cooling time or after muscle touch maneuver (40).

## Discussion

Thyroid surgery is one of the most commonly performed endocrine surgical procedures, the risk of complications is high due to the anatomical structure and physiological function of the RLN. Symptoms after thyroid surgery may include dysphagia, dysphonia, aspiration, choking, and even dyspnea (10–13). The management of symptomatic patients varied case by case, from voice therapy, vocal fold injection, thyroplasty to tracheotomy. It is noteworthy that patients who underwent thyroidectomy without RLN injury may also suffered from dysphonia and dysphagia. However, most of the symptoms are transient with good prognosis (9, 11–13). Avoiding iatrogenic RLN injury is essential for voice and swallowing outcomes after thyroid surgery. Impaired RLN function and vocal fold movement are problematic complications that may lead to medicolegal litigation after thyroid surgery in the quality-of-life era (8, 9, 42, 43).

Because they are ideal for dissection and provide a hemostatic effect, EBDs are currently used in more than 60% of thyroid surgeries (1). However, the high temperatures generated by EBD blades raise the risk of unexpected iatrogenic RLN thermal injury. This article reviewed recently published medical literature relevant to safety parameters for EBD use in thyroid surgery. For various EBDs, **Table 1** summarizes the recent literature on optimal activation distances in terms of safety and cooling time. Surgeons should be familiar with the safety parameters of an EBD before using it in thyroid surgery and should follow standard procedures for hemostasis and dissection near the RLN to prevent iatrogenic RLN thermal injury and to improve the voice outcomes of patients.

**Table 1:** Summary of safety parameters of EBDs near the RLN during thyroid surgery.

Device/Generator (Power)	Activation distance (activation time)	Cooling time	Reference (all by porcine model)
**Monopolar Electrocautery (15W)**	5 mm (1 sec)	1 sec	(25)
**Bipolar Electrocautery (30W)**	3 mm (1 sec)	1 sec	(25)
**Advanced Bipolar Energy Based Device**			
LigaSure Small Jaw (LSJ)/	2 mm (2-4 sec)	2 sec or	(31)
ForceTriad energy platform (level2)		muscle touch (immediately)	
LigaSure Exact Dissector (LED)/	1 mm (2-4 sec)	2 sec or	(35)
ValleylabTM LS10 energy platform		muscle touch (immediately)	
**Ultrasonic Energy BasedDevice**			
Harmonic Focus (HF)/	1 mm (3-10 sec)	10 sec or	(37)
Ethicon Endo-Surgery Generator G11(level5)		muscle touch(2 sec)	
Harmonic Focus+ (HF+)/	1 mm (4.7 ± 1.1sec)		(27)
Ethicon Endo-Surgery Generator G11(level5)			
Harmonic ACE/	4 mm (10-25 sec)		(38)
Ethicon Endo-Surgery Generator G11(level5)			
Harmonic ACE+/	1 mm (5-20 sec)		(38)
Ethicon Endo-Surgery Generator G11(level5)			
Sonicision/	1mm (1.5-2 sec)		(39)
maximum power mode (55 kHz)			
**Hybrid Energy Based Device (Ultrasonic and Bipolar Energy)**			
Thunderbeat (TB)	3 mm (10 sec)		(4)
**Ferromagnetic Energy Based Device**			
FMwand/	2 mm (3 sec)	1 sec or	(35)
FMX G1 Generator (Max45)		muscle touch (immediately)	
FMsealer/	2 mm (3 sec)	1 sec or	(35)
FMX G1 Generator (Max3)		muscle touch (immediately)	

In the past two decades, IONM use in thyroid surgery has become well established and is increasingly accepted worldwide. Additionally, many studies have reported that continuous IONM by periodic vagal stimulation can be useful in high-risk procedure and enables corrective action to prevent RLN traction injuries (44–46). Unlike traction injury, however, thermal injury often occurs suddenly and unexpectedly. Since IONM may be inapplicable for early detection and prevention of thermal injuries, safe use of EBDs is more important than using IONM to identify thermal injuries retrospectively.

Recent experimental and clinical studies indicate that thermal injury to the RLN is more severe than mechanical injury (i.e., injury caused by traction, compression, etc.) to the RLN because thermal injury tends to cause irreversible changes in nerve function (17, 19). Additionally, visual identification of a thermal injury to the RLN is often difficult, and lateral thermal spread can occur even when the heat source does not make direct contact with the nerve (31, 37). Protein denaturation and RLN injuries occur at a temperature of 60°C (47). Thermal stimuli applied to the RLN at temperatures over 60°C can cause permanent functional damage to the endoneurium (19). Since most EBDs reach temperatures exceeding 60°C after activation, surgeons must carefully consider the risk of endoneurium injury, regardless of the EBD type used. Maximum activation temperatures of ultrasonic EBDs and hybrid devices (e.g. TB) may exceed 200°C, only lower to monopolar electrocautery which have a higher maximum activation temperature (> 350°C) (28, 29, 33, 39). Advanced bipolar EBDs, bipolar electrocautery and FMsealer have maximum activation temperatures ranging from 80 to 100°C (33, 41, 48). Therefore, surgeons should remain cognizant that using EBDs for dissection near the RLN can potentially cause thermal injury of varying severity, especially during endoscopic procedures in which the surgical field is limited and the EBDs is commonly used for dissection.

Different EBDs deliver energy through different mechanisms, and the high temperature generated by an EBD may not be proportional to the activation distance (32). Recent studies also indicate that advanced bipolar devices induce greater thermal spread compared to ultrasonic devices. However, the temperature of adjacent tissue affected by thermal spread reportedly remains below 30°C, which would not substantially change EMG (39, 48).

Notably, Hayami et al. argued that activating EBDs in the wet condition (e.g., after exposure to liquid content from tissue) generates high-temperature steam. Since the steam, hyperthermal liquid, or smog may cause thermal injury to the RLN, caution should be taken when operate in specific condition, and increasing the activation distance is essential (49, 50).

Long duration of EBDs use may cause tissue shrinkage or thermal injury (38). Temperatures potentially generated by EBDs increase as activation time increases. Heat production is slower in ultrasonic EBDs compared to electrocautery equipment. Ultrasonic EBDs, temperatures may reach 150 to 200°C when activation time exceeds 10 seconds (29, 33, 39).

Similarly, advanced bipolar EBDs reach much higher temperatures after a double activation compared to a single activation. Consequently, thermal injuries may occur when an advanced bipolar EBDs is not allowed to cool between activations (35).

For some EBDs, data for cooling time were unavailable and were not included in **Table 1**. Experiments using infrared cameras and thermo-sensors had elucidated this issue. According to the literature, the time required for ultrasonic EBDs to cool to 60°C is almost two-fold longer than that for advanced bipolar EBDs (32–34). The surgeons may opt to perform muscle touch maneuver if the surgeons concludes that the long cooling time for an EBD during surgery would raise the risk of an extended surgical time. For ultrasonic EBDs, the recommended minimum duration of the muscle touch maneuver is 2 seconds (37).

This article reviewed relevant medical literature on the safety parameters for use of EBDs in thyroid surgery, including minimum safe distance from the RLN and the cooling duration of EBDs. Applying continuous IONM in animal experiments is an ideal method to establish the safety parameters of newly launched EBDs (51). Notably, all reviewed studies were animal studies, and all used porcine models of the RLN since experimentally inducing RLN injury in a human patient would violate ethical guidelines. Recommended distances for safe use of EBDs slightly differed among some studies, possibly due to differences in the mechanical properties of tissue specimens (e.g. wet versus dry condition), differences in experimental animal species, and differences in instrument settings and methodologies. Neural and tissue characteristics may differ even when the same animal model is used (37, 49). Therefore, data obtained in animal experiments should be applied cautiously to human patients (30). Future large observational clinical studies are needed for further verification of the findings of this study.

## Conclusion

In conclusion, energy-based device (EBD) in thyroidectomy yields many superior outcomes, including considerable reduction of operative time, incision length, blood loss, and post-operative pain. One major advantage is significantly lowered rate of postoperative neck hematoma and postoperative hypocalcemia. To avoid an inadvertent iatrogenic RLN thermal injury caused by EBDs, standard procedures for safe use of these advanced medical devices must be developed and implemented. Many studies agree that animal models are ideal for experiments in continuous IONM because they provide objective data that can be used for electrophysiological evaluation of RLN thermal injury and for development of safety parameters for newly developed EBDs.

In general, conventional electrocautery generates higher temperature and wider thermal spread range, but when applying EBDs near the RLN adequate activation distance and cooling time are still required to avoid inadvertent thermal injury. Understanding EBD safety parameters and following standard procedures for using EBDs in thyroid surgery can improve safety and surgical outcomes, especially in voice quality and vocal cord mobility.

## Author Contributions

JJ-W, TY-H, and CW-W conceived and designed the study. Administrative support was obtained by CH-L, LF-W. Provision of study materials by IC-L, PY-C, HC-C, HY-C, HY-T, YC-L and G-D. had collected and assembled the data. Data analysis and interpretation was done by FY-C, TY-H, CW-W. All authors were participated in manuscript writing and final approval of manuscript.

## Funding

This study was supported by grants from Kaohsiung Medical University Hospital (KMUH109-9M44), Kaohsiung Municipal Siaogang Hospital/Kaohsiung Medical University Research Center grants (KMHK-DK (C)110009, I-109-04, H-109-05, I-108-02) and Ministry of Science and Technology (MOST 108-2628-B-037-006, MOST 109-2628-B-037-014, MOST 110-2314-B-037-104-MY2, MOST 110-2314-B-037-120), Taiwan.

## Conflict of Interest

The authors declare that the research was conducted in the absence of any commercial or financial relationships that could be construed as a potential conflict of interest.

## Publisher’s Note

All claims expressed in this article are solely those of the authors and do not necessarily represent those of their affiliated organizations, or those of the publisher, the editors and the reviewers. Any product that may be evaluated in this article, or claim that may be made by its manufacturer, is not guaranteed or endorsed by the publisher.
